# Geographic Liver

**DOI:** 10.7759/cureus.45563

**Published:** 2023-09-19

**Authors:** Ariya Natarajan, Anjali R Daniel, Rohan K Mangal, Thor S Stead, Latha Ganti

**Affiliations:** 1 Biomedical Sciences, University of Central Florida, Orlando, USA; 2 Biology, Emory University, Atlanta, USA; 3 Medicine, University of Miami Miller School of Medicine, Miami, USA; 4 Medicine, The Warren Alpert Medical School of Brown University, Providence, USA; 5 Medical Sciences, The Warren Alpert Medical School of Brown University, Providence, USA; 6 Emergency Medicine & Neurology, University of Central Florida College of Medicine, Orlando, USA

**Keywords:** masld, fatty liver, hepatic steatosis (masld), metabolic dysfunction-associated steatohepatitis (mash), geographic liver

## Abstract

The authors present the case of a man with a relatively benign clinical presentation who had a computed tomography scan that revealed a "geographic liver" pattern. The radiologic appearance of hepatic steatosis, its significance, and its association with metabolic syndrome highlight the importance of this radiologic finding.

## Introduction

Hepatic steatosis is a common clinical entity, defined as intrahepatic fat greater than 5% [1]. Geographic liver, also known as geographic liver steatosis, results from the irregular deposition of fat, in contrast to general hepatic steatosis, where the fat distribution is more generalized. This entity is easily recognized in ultrasonography and computed tomography. The pattern is distinguished from a focal liver lesion by noting that it does not produce any mass effect on adjacent structures [2]. Hepatic steatosis can be seen with both nonalcoholic liver disease as well as nonalcoholic steatohepatitis. It is estimated that 20 to 30 percent of the adult population in developed countries have nonalcoholic fatty liver disease and that it is becoming more prevalent in children as well [1,2]. Risk factors for hepatic steatosis include obesity, diabetes or insulin resistance, dyslipidemia, and hypertension [3]. The condition is usually asymptomatic, but, in some cases, can cause abdominal pain, nausea, swollen legs, and extreme tiredness and mental confusion. Like geographic tongue [4] geographic liver derives its name from the appearance of the liver on abdominal CT scans, resembling borders of continents that are on a map [5].

## Case presentation

**Figure 1:**
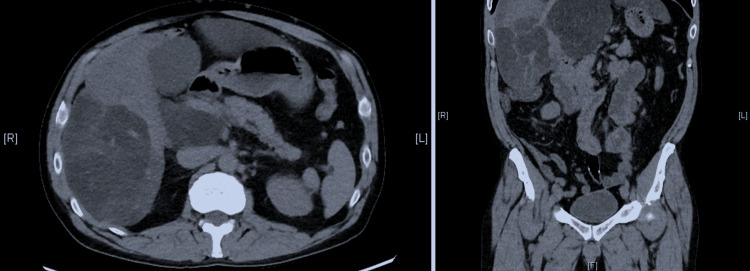
Axial (left panel) and sagittal (right panel) views of the liver on a non-contrast abdominal computed tomography scan, demonstrating hypoattenuated patches in the liver, the "geographic liver" pattern.

A 45-year-old male presented to the emergency department with fatigue, occasional nausea, and intermittent vague abdominal pain. The patient denied any past medical or surgical history. He took no medications on a daily basis and denied any allergies to medications. He was a nondrinker and a nonsmoker. Vital signs were temperature 97.8^0^F, pulse 83 beats per minute, blood pressure 143/89 mmHg, respirations 17 per minute, and pulse oximetry 98% on room air. He was afebrile, alert, and oriented. Physical exam demonstrated an overweight man (BMI 31) with a large abdominal girth of 41 inches. Hepatomegaly without tenderness was noted on the physical exam. There were no signs of cirrhosis present. Laboratory analysis revealed a blood glucose of 170 mg/dL (non-fasting), normal complete blood count, and chemistries. Liver enzymes were mildly elevated; alanine transaminase (ALT) 41 U/L, aspartate transaminase (AST) 37 U/L, gamma-glutamyl transferase 15 U/L, and alkaline phosphatase 100 U/L. The fasting lipid profile revealed a high-density lipoprotein cholesterol of 34 mg/dL, triglyceride levels of 180 mg/dL, and a fasting blood sugar of 130 mg/dL. The patient underwent computed tomography scanning of his abdomen and pelvis, which revealed a “geographic liver” pattern with patchy areas that look like separate continents, due to hepatic steatosis (Figure 1).

**Figure 2:**
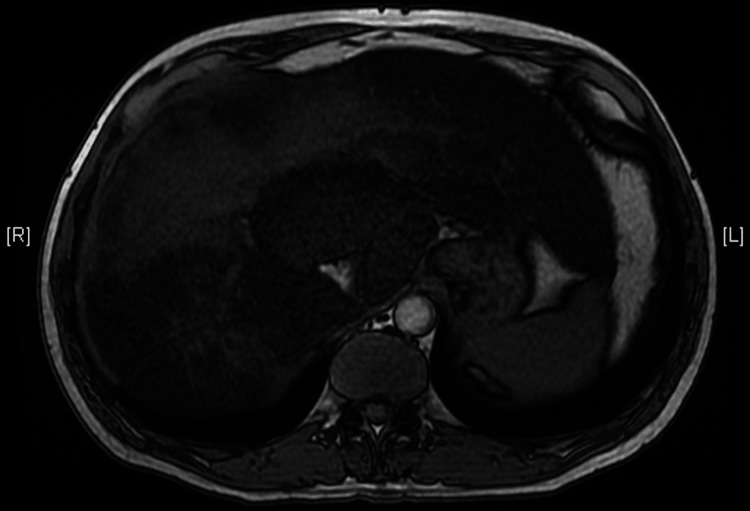
Magnetic resonance axial image demonstrating a patchy "geographic" appearance of the liver.

Magnetic resonance imaging demonstrated a similar pattern (Figure 2).

A working diagnosis of metabolic dysfunction-associated steatotic liver disease (MASLD) was made. MASLD is considered to be the hepatic manifestation of the metabolic syndrome. 

## Discussion

MASLD replaces the previous term nonalcoholic fatty liver disease (NAFLD). MASLD encompasses patients who have hepatic steatosis and have at least one of five cardiometabolic risk factors [6]. MASLD is a common liver condition characterized by the accumulation of fat in hepatocytes in the absence of significant alcohol consumption. When MASLD progresses to include inflammation and hepatocellular injury, it is referred to as metabolic dysfunction-associated steatohepatitis (MASH) [6]. The previous term for MASH was nonalcoholic steatohepatitis (NASH). Approximately 25-40% of patients with MASH will eventually develop liver fibrosis and ultimately cirrhosis [7]. MASLD and MASH have become a global public health concern due to their increasing prevalence. It is estimated that approximately 10-30% of the global population has MASLD [8]. This estimate rises to as high as 57-74% of those who are obese [9]. The prevalence is higher in Western countries and is strongly associated with obesity and metabolic syndrome. Criteria for metabolic syndrome vary by organization. The National Cholesterol Education Program Adult Treatment Panel III (NCEP-ATP III) defines metabolic syndrome in men as the presence of any three of the following five criteria: waist circumference of 102 cm (40 inches) for men; triglyceride levels >150 mg/dL, HDL cholesterol <40 mg/dL, blood pressure >130/85 mmHg, and a fasting blood sugar >100 mg/dL, or being on medication for diabetes [10]. Our patient clearly met the criteria for metabolic syndrome.

The differential diagnosis for MASH is broad and encompasses any condition where hepatic steatosis and fibrosis can occur, including alcoholic liver disease, viral hepatitis, autoimmune hepatitis [11], hemochromatosis [12], Wilson's disease [13], and medication or toxin-induced liver injury.

## Conclusions

The term geographic liver refers to the fatty deposits in the liver that give it an irregular pattern on imaging. While hepatic steatosis by itself is a benign condition, it can lead to increased morbidity when accompanied by inflammation. Recognition of this imaging pattern can help physicians have conversations with their patients about lifestyle choices and the risk of metabolic dysfunction.
